# Temperature differentially affects subsequent layers of auditory neurons in the locust

**DOI:** 10.1186/1471-2202-12-S1-P287

**Published:** 2011-07-18

**Authors:** Frederic A Roemschied, Monika JB Eberhard, Bernhard Ronacher, Susanne Schreiber

**Affiliations:** 1 Institute for Theoretical Biology, Humboldt-Universität zu Berlin, 10115 Berlin, Germany; 2 Bernstein Center for Computational Neuroscience Berlin, Humboldt-Universität zu Berlin, 10115 Berlin, Germany; 3 Department of Biology, Humboldt-Universität zu Berlin, 10115 Berlin, Germany

## 

Temperature influences basic properties of nerve cells such as spike rate, conduction velocity, and spike amplitude. This is relevant for ectothermic animals whose body temperature changes with ambient temperature. Here, we investigate the effect of temperature on signal processing in the grasshopper acoustic communication system. For these insects, the decoding of temporal characteristics of conspecific calls is crucial for mate recognition and may be impaired by temperature differences between sender and receiver.

The peripheral auditory system is located within the metathoracic ganglion, where the first steps of song pattern recognition and analysis of sound direction are accomplished. Receptor neurons, local interneurons, and ascending neurons constitute these first three processing stages, forming a hierarchically organized feed-forward network. Previous studies revealed an improvement of temporal resolution at higher temperatures due to a higher precision of spike timing. However, neurons of the three processing stages were not equally affected by variation in temperature.

In the present study, responses of locust auditory receptors, local interneurons and ascending neurons to short acoustic broad-band noise stimuli of various intensities were recorded intracellularly at a set of behaviorally relevant temperatures. Based on these data, temperature coefficients (Q_10_) were determined for the intensity-response curves of all neurons.

Our results confirm an influence of temperature on spike count, shape, and duration, as well as first spike latencies. However, the overall response pattern and the shape of the intensity-response curve varied less than expected. In particular, receptor neurons and ascending neurons exhibited lower Q_10_ values than local interneurons. We conclude that distinct mechanisms of temperature compensation are present at subsequent processing stages.

To understand these phenomena, we reproduced the observed electrophysiological responses at each processing stage using conductance-based neuronal models. We then investigated how the observed temperature compensation can arise from specific cell-intrinsic mechanisms or, alternatively, from the network structure of the metathoracic auditory pathway.

